# GenAI dependence and graduate students’ research creativity: the roles of critical thinking and human–AI collaboration quality

**DOI:** 10.3389/fpsyg.2026.1896963

**Published:** 2026-09-23

**Authors:** Shi Yin, Ruqiao Wu, Xuejun Liu, Renjie Li

**Affiliations:** 1 School of Law, Chongqing University, Chongqing, China; 2 Institute of Education, Xiamen University, Xiamen, China; 3 Faculty of Humanities and Social Sciences, Doctoral School of Education, University of Pécs, Pécs, Hungary

**Keywords:** critical thinking, existential dependence, functional dependence, generative artificial intelligence, human-AI collaboration quality, research creativity

## Abstract

**Introduction:**

As generative artificial intelligence (GenAI) becomes increasingly integrated into graduate students’ academic research, the relationships between GenAI dependence and research creativity have attracted growing scholarly attention. This study distinguishes task-oriented functional dependence from deeper psychological existential dependence and examines their associations with research creativity to examine their associations with research creativity, as well as the potential mediating roles of critical thinking and human–AI collaboration quality.

**Methods:**

Questionnaire data were collected from 1,157 graduate students recruited through online convenience sampling. Structural equation modeling (SEM) was used to examine the overall structural framework linking GenAI dependence, critical thinking, human–AI collaboration quality, and research creativity. PROCESS Model 6 with 5,000 bootstrap resamples was subsequently used to examine and compare the indirect pathways associated with functional and existential dependence while controlling for demographic variables.

**Results:**

The results indicated that: (1) Functional dependence and existential dependence were both positively associated with research creativity, with the total association for functional dependence (β = 0.457) being substantially stronger than that for existential dependence (β = 0.147); (2) critical thinking was significantly associated with the relationship between GenAI dependence and research creativity; (3) human-AI collaboration quality was also significantly associated with research creativity; (4) critical thinking and human-AI collaboration quality jointly constituted a significant serial mediation pathway linking GenAI dependence and research creativity.

**Discussion:**

These findings contribute to the literature on GenAI use and graduate education by highlighting the differentiated associations of functional and existential dependence with research creativity. The findings also suggest that critical thinking and high-quality human–AI collaboration may be important factors associated with graduate students’ self-reported research creativity. Given the cross-sectional design of the study, the findings should be interpreted as associative rather than causal.

## 1 Introduction

Generative artificial intelligence (GenAI), represented by large language models such as ChatGPT, has rapidly become integrated into higher education and academic research. For graduate students, GenAI is no longer merely an auxiliary writing tool; it can assist with literature retrieval, idea generation, data interpretation, academic writing, feedback seeking, and methodological learning. Existing studies in educational technology and information systems suggest that large language models may expand access to learning resources, personalize explanations, and improve the efficiency of knowledge-related tasks, while also raising concerns regarding inaccurate outputs, algorithmic opacity, academic integrity, and the possibility of reduced independent cognitive engagement among learners (Dwivedi et al., 2023; Kasneci et al., 2023; Yan et al., 2024). Accordingly, the key issue is not simply whether graduate students use GenAI, but how different patterns of dependence on GenAI are associated with higher-order academic outcomes such as research creativity.

Research creativity is widely regarded as one of the core outcomes of graduate education. Unlike routine academic performance, research creativity involves identifying meaningful problems, connecting distant ideas, evaluating competing explanations, and transforming knowledge into original scholarly contributions. Creativity research has consistently emphasized that creative performance depends not only on domain-specific knowledge but also on cognitive processes such as flexible thinking, intrinsic motivation, problem representation, and critical evaluation (Amabile, 1983; Kaufman and Beghetto, 2009). In the context of GenAI use, these cognitive processes may become particularly relevant. Interacting with GenAI may expose students to abundant and diverse suggestions, potentially broadening idea exploration and supporting divergent thinking. At the same time, excessive reliance on AI-generated responses may also be associated with reduced independent exploration, superficial synthesis, or lower distinctiveness of individual ideas. Recent studies further suggest that the relationship between AI use and creativity is not uniform or automatic, but may depend on how users evaluate, select, and integrate AI-generated outputs into their own thinking processes (Lee and Chung, 2024).

One reason for these mixed possibilities may be that GenAI reliance is not a unitary phenomenon. Although prior research has extensively examined technology adoption and acceptance (Venkatesh et al., 2003), comparatively less attention has been paid to qualitatively different forms of GenAI dependence in academic research. Recent scale-development research distinguishes between functional dependence and existential dependence (Li et al., 2025). Functional dependence refers to task-oriented and instrumental reliance on GenAI to improve efficiency and support specific research activities, whereas existential dependence reflects a deeper psychological and cognitive reliance in which users may feel less confident, autonomous, or capable without AI assistance. This distinction is theoretically important because similar levels of GenAI use may reflect different psychological orientations and may therefore show different associations with research creativity. Treating GenAI dependence as a differentiated rather than unitary construct may thus provide a more precise account of how AI reliance is related to graduate students’ research development.

From this perspective, functional dependence may reflect a more strategic and instrumental pattern of GenAI use in academic research. Graduate students who report higher functional dependence may use GenAI to assist with repetitive or procedural tasks while remaining actively engaged in higher-order activities such as problem framing, theoretical reasoning, and idea integration. By contrast, existential dependence may reflect a stronger psychological reliance on AI-generated support in ways that are associated with lower levels of independent evaluation and autonomous academic judgment. Prior studies on AI text generators in higher education further suggest that students’ critical thinking experiences may vary depending on whether learners critically evaluate AI-generated outputs or treat them as authoritative responses (Essien et al., 2024; Klingbeil et al., 2024). Therefore, distinguishing between functional dependence and existential dependence may provide a more nuanced framework for understanding the heterogeneous associations between GenAI dependence and research creativity.

Beyond distinguishing forms of dependence, the relationship between GenAI dependence and research creativity may also be associated with how students cognitively evaluate and collaboratively engage with AI. Critical thinking enables graduate students to question, verify, and selectively integrate AI-generated information rather than accepting it uncritically (Halpern, 1998; Suriano et al., 2025). Human–AI collaboration quality, in turn, reflects the extent to which users interact with AI in reflective, iterative, and complementary ways, including formulating appropriate prompts, evaluating feedback, refining questions, and integrating useful outputs into their own academic work (Nguyen et al., 2024). These two factors may also be statistically linked: students who engage more critically with AI-generated information may report higher-quality patterns of human–AI collaboration, which may in turn be associated with more effective integration of AI-supported information into research activities. Accordingly, critical thinking and human–AI collaboration quality are examined as both independent and sequential statistical pathways linking GenAI dependence with research creativity. Given the cross-sectional design, these pathways are interpreted as patterns of association rather than evidence of a causal psychological process.

Despite the growing literature on GenAI in education, several important gaps remain. First, many existing studies treat AI use or AI reliance as a unitary construct, which may overlook the potentially different associations of functional dependence and existential dependence with academic outcomes. Second, prior research has frequently focused on general academic performance, writing efficiency, or ethical concerns, whereas the relationships between GenAI dependence and graduate students’ research creativity remain relatively underexplored. Third, relatively few studies have simultaneously examined critical thinking and human–AI collaboration quality as theoretically relevant factors associated with GenAI use, despite their potential importance for reflective and creative academic engagement. Addressing these gaps is particularly important in graduate education because research creativity may depend not only on access to advanced technological tools but also on students’ ability to critically evaluate and meaningfully integrate AI-generated information into their own research processes.

Accordingly, this study has three objectives: (1) to examine the associations between GenAI dependence and graduate students’ research creativity while distinguishing functional dependence from existential dependence; (2) to examine the statistical indirect roles of critical thinking and human–AI collaboration quality in these associations; and (3) to compare the association patterns and indirect pathways corresponding to functional and existential dependence. Drawing on creativity theory, critical thinking research, and the human–AI collaboration literature, the study proposes and tests a serial mediation framework linking GenAI dependence, critical thinking, human–AI collaboration quality, and research creativity.

The study makes three contributions. First, it advances a differentiated conceptualization of GenAI dependence by distinguishing functional from existential dependence rather than treating AI reliance as a homogeneous construct. Second, it integrates critical thinking and human–AI collaboration quality within a common analytical framework, thereby extending understanding of the cognitive and collaborative correlates of GenAI dependence and research creativity. Third, it provides practical implications for graduate AI literacy education by highlighting the importance of critical evaluation and reflective human–AI collaboration alongside the effective use of GenAI tools.

## 2 Literature review and research hypotheses

### 2.1 The relationship between GenAI dependence and research creativity

GenAI dependence refers to an individual’s tendency to develop relatively stable reliance on artificial intelligence tools at the cognitive, emotional, or behavioral levels, and its theoretical foundations can be traced to research on internet addiction and smartphone addiction (Al-Obaydi and Pikhart, 2026). With the widespread adoption of generative artificial intelligence in higher education and academic research, GenAI dependence has gradually emerged as an important topic in educational psychology. Compared with general forms of technology dependence, GenAI dependence may involve more extensive cognitive engagement because GenAI systems are capable not only of executing operational tasks but also of generating content, providing suggestions, simulating dialogue, and supporting idea development (Chen et al., 2026).

At present, there is no unified consensus regarding the conceptualization of GenAI dependence. Drawing on existing research, the present study conceptualizes graduate students’ GenAI dependence as comprising two dimensions, functional dependence and existential dependence. Functional dependence refers to the instrumental and task-oriented use of GenAI to support specific academic or research-related activities. Existential dependence, by contrast, refers to a deeper psychological and cognitive reliance on GenAI, in which individuals may feel less confident or less capable of completing research tasks independently without AI assistance. The relationship between the two is that functional dependence represents an objective description at the behavioral level of use, whereas existential dependence represents deep involvement at the psychological level (Li et al., 2025). Existing research has shown that functional dependence and existential dependence have different antecedents and consequences, which lays the foundation for treating them as distinct constructs in subsequent studies.

Clark and Chalmers (1998), in their Extended Mind Theory, proposed that when an external tool forms a stable and seamless coupling relationship with an individual, the tool can be regarded as part of the individual‘s cognitive system (Hernández-Orallo, 2025). According to this perspective, external technological tools may become integrated into individuals’ cognitive activities when interactions between humans and technologies become sufficiently stable and embedded. In the context of graduate research, GenAI may therefore function not merely as an external technological aid but also as a cognitive support resource within broader research practices. Prior studies suggest that graduate students may use GenAI to assist with activities such as information retrieval, text organization, preliminary analysis, and idea exploration, potentially allowing greater attention to be allocated to higher-order academic tasks, including theoretical reasoning, hypothesis development, and research design (Xie et al., 2026). In other words, moderate dependence on GenAI does not mean abandoning thinking; rather, it involves outsourcing lower-order cognitive tasks to GenAI to achieve a strategic reallocation of cognitive resources. This is the core logic of the empowerment perspective. Within this perspective, functional dependence represents instrumental rationality, where individuals maintain active control and choice over GenAI. Existential dependence, while carrying the risk of psychological attachment, may also yield cognitive benefits through frequent, habitual use of GenAI, as such use provides more opportunities to access diverse perspectives and interdisciplinary connections generated by GenAI, thereby obtaining richer knowledge input and creative stimulation.

### 2.2 The mediating role of critical thinking

Critical thinking generally refers to an individual’s cognitive disposition and capacity to question, analyze, evaluate, and reflect upon information. It is widely regarded as a core objective of higher education and a hallmark outcome of cognitive development. Its importance is particularly pronounced in the age of AI (Larson et al., 2024). In the era of generative artificial intelligence, critical thinking has acquired renewed importance because students increasingly interact with AI-generated information that may appear coherent and authoritative while still containing inaccuracies, oversimplifications, or algorithmic biases. A recent systematic literature review suggested that critical thinking in AI-supported contexts should be understood as a form of metacognitive regulation involving the evaluation of AI-generated outputs, the identification of potential biases, and the adjustment of one’s own cognitive strategies accordingly (Hyytinen et al., 2025). Within graduate research contexts, critical thinking may be reflected in students’ active examination, questioning, and selective integration of AI-generated content rather than passive acceptance of AI responses. Prior studies further indicate that AI self-regulation strategies are associated with higher levels of critical thinking in AI-assisted learning contexts, suggesting that critical thinking may play an important role in shaping how students engage with AI-generated information (Raitskaya and Tikhonova, 2025). Existing research has confirmed that critical thinking is not only an outcome that can be cultivated in AI-assisted learning but also a significant condition that may influence learning outcomes.

Amabile’s componential theory of creativity suggests that domain-relevant knowledge and creativity-relevant skills are central components of creative performance (Amabile, 1983; Conti et al., 1996). Critical thinking is precisely a key creativity-relevant skill. It enables individuals, after generating numerous ideas through divergent thinking, to perform convergent screening through logical reasoning and evidence evaluation, thereby refining innovative outcomes that are both novel and scientifically valuable. In the context of graduate research, critical thinking helps researchers identify theoretical gaps during the literature review stage, evaluate the feasibility of research designs during the methodology stage, judge the reliability of results during the data analysis stage, and construct persuasive argumentative logic during the academic writing stage (Mahanal et al., 2019). Research indicates that in the application of AI technology, critical thinking enables students to maintain questioning and reflection while rapidly accessing information (Darwin et al., 2024).

### 2.3 The mediating role of human-AI collaboration quality

Large language model-based GenAI systems have increasingly moved beyond functioning solely as simple command–response tools and are now frequently used in more interactive and iterative ways during academic work. This fundamental change shifts the human-GenAI relationship from usage to collaboration, wherein individuals and GenAI form a joint collaborative unit to tackle complex cognitive tasks together. Thus, the focus turns to the quality of outcomes produced through human-AI collaboration (Kunz et al., 2025). Cognitive synergy theory emphasizes that human-AI collaboration is not merely a simple division of labor but rather a form of complementary division. Humans excel at abstract reasoning, value judgment, and creative association, whereas AI excels at information integration, pattern recognition, and large-scale data processing. When the two form a high-quality collaborative relationship, AI-generated outputs can break graduate students existing thinking patterns and stimulate new research perspectives and ideas (Ren et al., 2023). At the same time, studies have also reported important limitations of AI-generated academic content. Importantly, evidence regarding AI-supported academic performance is not uniformly positive. For example, AI-generated manuscripts have been found to score relatively higher in readability and structural clarity while demonstrating comparatively lower levels of technical accuracy and analytical depth (Nathani et al., 2025). These findings suggest that the quality of outcomes associated with GenAI use may depend not only on AI-generated responses themselves but also on how students critically evaluate and meaningfully integrate AI-generated information into their own academic work. In graduate research contexts, higher-quality human–AI collaboration may therefore be associated with more reflective engagement with AI-generated outputs, more effective integration of disciplinary knowledge and AI-supported information, and more active participation in idea refinement and research development. By contrast, lower-quality collaboration may be associated with more passive acceptance of AI-generated responses or less critical evaluation of AI-supported content. Accordingly, human–AI collaboration quality may help explain the relationship between GenAI dependence and graduate students’ research creativity.

### 2.4 The serial mediating role of critical thinking and human-AI collaboration quality

Critical thinking is an important prerequisite for human-AI collaboration quality. First, critical thinking helps graduate students accurately assess the quality and limitations of AI-generated outputs, enabling them to adjust their interaction strategies, which directly enhances the fluency and effectiveness of human-AI collaboration. Second, critical thinking enables graduate students to actively construct problems and decompose tasks, rather than passively accepting AI-generated answers, thereby forming a collaborative model in which humans take the lead and AI serves as an auxiliary tool. Finally, critical thinking strengthens graduate students’ calibrated trust in GenAI neither over-trusting nor over-skeptical and such appropriate human-AI trust serves as the foundation for high-quality collaboration. Existing research has shown that individuals with higher levels of critical thinking demonstrate greater strategic flexibility and superior task performance when interacting with AI (Larson et al., 2024; Yusuf et al., 2024).

Synthesizing the above arguments, the present study proposes a serial mediation framework in which critical thinking and human–AI collaboration quality constitute sequential statistical pathways linking GenAI dependence with research creativity. Higher levels of functional dependence may be associated with greater engagement in AI-supported research activities, while critical thinking may be associated with more reflective evaluation of AI-generated information and, in turn, higher-quality human–AI collaboration. Higher-quality collaboration may subsequently be associated with greater self-reported research creativity. A similar indirect pattern is examined for existential dependence, although the nature and magnitude of these associations may differ because existential dependence reflects a deeper form of psychological reliance on GenAI.

Given the cross-sectional design, the proposed sequence represents a theoretically informed statistical mediation model rather than evidence that GenAI dependence causally changes critical thinking, collaboration quality, or research creativity.

### 2.5 Research hypotheses

Accordingly, the following hypotheses are proposed:

H1: GenAI dependence is positively associated with graduate students’ research creativity.
H1a: Functional dependence is positively associated with graduate students’ research creativity.
H1b: Existential dependence is positively associated with graduate students’ research creativity.
H2: Critical thinking constitutes a significant indirect pathway linking GenAI dependence with research creativity.
H2a: Critical thinking constitutes a significant indirect pathway linking functional dependence with research creativity.
H2b: Critical thinking constitutes a significant indirect pathway linking existential dependence with research creativity.
H3: Human–AI collaboration quality constitutes a significant indirect pathway linking GenAI dependence with research creativity.
H3a: Human–AI collaboration quality constitutes a significant indirect pathway linking functional dependence with research creativity.
H3b: Human–AI collaboration quality constitutes a significant indirect pathway linking existential dependence with research creativity.
H4: Critical thinking and human–AI collaboration quality constitute a significant serial indirect pathway linking GenAI dependence with research creativity.
H4a: Critical thinking and human–AI collaboration quality constitute a significant serial indirect pathway linking functional dependence with research creativity.
H4b: Critical thinking and human–AI collaboration quality constitute a significant serial indirect pathway linking existential dependence with research creativity.

## 3 Research method

### 3.1 Sample

This study employed an online convenience sampling approach to recruit graduate students in China. The questionnaire was administered through Wenjuanxing, a widely used online survey platform in China, and was distributed through university-based WeChat groups and social media platforms. Participation was voluntary. A total of 1,277 questionnaires were collected. After excluding invalid responses, including those exhibiting patterned answering, implausibly short or long completion times, or failure to pass attention-check items, 1,157 valid questionnaires were retained, corresponding to a valid-response rate of 90.60%. Before accessing the questionnaire, participants were provided with information about the study purpose, procedures, voluntary nature of participation, confidentiality, and intended use of the data. Participants were required to indicate their agreement by selecting “I agree to participate in this study” on the first page of the online questionnaire before proceeding to the survey items. As all participants were graduate students aged 18 or above and capable of making informed judgments, they were provided with comprehensive information regarding the survey‘s objectives, procedures, and data usage before participation, and informed consent was obtained prior to enrollment. This sample size surpasses the 5:1 parameter-to-respondent ratio recommended for SEM (Bentler and Chou, 1987), ensuring statistical robustness. The valid sample characteristics are presented in Table 1.

**TABLE 1:** Valid sample characteristics.

Variable	Category	*N*	Percentage (%)
Gender	Male	553	47.796
	Female	604	52.204
Degree type	Academic master	401	34.659
	Professional master	523	45.203
	Academic doctor	175	15.125
	Professional doctor	58	5.013
Grade	First year	450	38.894
	Second year	332	28.695
	Third year	255	22.040
	Fourth year	96	8.297
	Other grade	24	2.074
Discipline	Humanities and arts	121	10.458
	Social sciences and law	228	19.706
	Natural sciences	235	20.311
	Engineering	261	22.558
	Agriculture	70	6.050
	Medicine	153	13.224
	Interdisciplinary/ emerging fields	50	4.322
	Other disciplines	39	3.371

### 3.2 Scale

Drawing on the AI Dependence Questionnaire developed by Li et al. (2025), the scale comprised 18 items covering two dimensions: functional dependence and existential dependence. All items were rated on a 5-point Likert scale ranging from 1 (“strongly disagree”) to 5 (“strongly agree”), with higher scores indicating higher levels of individual AI dependence. In this study, the Cronbach‘s α coefficient of this scale was 0.913. The confirmatory factor analysis (CFA) results indicated a good fit: χ^2^ = 676.92, df = 134, CFI = 0.95, TLI = 0.95, RMR = 0.05.

Drawing on the study by Acosta-Enriquez et al. (2025), the Critical Thinking Questionnaire developed by Acosta-Enriquez et al. was adopted. The scale comprised five items, all rated on a 5-point Likert scale ranging from 1 (“strongly disagree”) to 5 (“strongly agree”), with higher scores indicating higher levels of critical thinking (Zhang et al., 2024). In this study, the Cronbach‘s α coefficient of this scale was 0.863. The confirmatory factor analysis (CFA) results indicated a good fit: χ^2^ = 71.12, df = 5, CFI = 0.97, TLI = 0.95, RMR = 0.03.

The Human-AI Collaboration Quality Questionnaire developed by Shang et al. (2026) was adopted. The scale comprises 11 items covering three dimensions: outcome quality, comfort, and efficiency. All items were rated on a 5-point Likert scale ranging from 1 (“strongly disagree”) to 5 (“strongly agree”), with higher scores indicating higher levels of human-AI collaboration quality (Shang et al., 2026). In this study, the Cronbach‘s α coefficient of this scale was 0.935. The confirmatory factor analysis (CFA) results indicated a good fit: χ^2^ = 537.360, df = 41, CFI = 0.94, TLI = 0.92, RMR = 0.04.

The Graduate Research Creativity Scale, adapted from the creativity scale developed by Zhang et al. (2023), was adopted. The scale comprises six items, all rated on a 5-point Likert scale ranging from 1 (“strongly disagree”) to 5 (“strongly agree”), with higher scores indicating higher levels of graduate students “research creativity. In this study, the Cronbach’s α coefficient of this scale was 0.885. The confirmatory factor analysis (CFA) results indicated a good fit: χ^2^ = 53.60, df = 9, CFI = 0.99, TLI = 0.98, RMR = 0.02.

### 3.3 Data analysis

SPSS 26.0 was used to conduct common method bias testing, descriptive statistics, and correlation analysis. Amos 24 was used to perform confirmatory factor analysis (CFA) to evaluate the construct validity of the measurement model and further test common method bias through the single-factor CFA approach. In addition, structural equation modeling (SEM) was conducted to examine the overall fit of the proposed theoretical framework and the structural relationships among the study variables. Furthermore, the PROCESS macro for SPSS (Model 6) was employed to test the serial mediating effects of critical thinking and human-AI collaboration quality. Bias-corrected bootstrap confidence intervals based on 5,000 resamples were used to estimate the indirect effects. Gender, degree type, grade level, and academic discipline were included as control variables in all mediation analyses.

## 4 Research results

### 4.1 Common method bias test

The Harman one-factor test was conducted to examine common method bias. The results showed that five factors with eigenvalues greater than one were extracted without rotation, and the first factor accounted for 34.119% of the total variance, which is below the critical threshold of 40% (Fuller et al., 2016). In addition, confirmatory factor analysis (CFA) was conducted to test for common method bias by loading all measurement items onto a single common factor to construct a one-factor model. The model fit results were as follows: χ^2^/df = 9.812, GFI = 0.633, AGFI = 0.590, CFI = 0.782, RMR = 0.143, NFI = 0.763. None of the fit indices met the recommended criteria for good fit, indicating that a single factor could not explain most of the variance. Therefore, the results suggest that common method bias was unlikely to pose a serious threat to the validity of the findings.

### 4.2 Descriptive statistics and correlation analysis

The descriptive statistics and correlation analyses of the study variables are presented in Table 2. The mean scores of all variables ranged from 3.11 to 3.77 (measured on a 5-point Likert scale), indicating that graduate students’ GenAI dependence, critical thinking, human-AI collaboration quality, and research creativity were all at moderately high levels. Significant positive correlations were observed among all study variables. Specifically, functional dependence was significantly positively correlated with existential dependence (*r* = 0.339, *p* < 0.01), critical thinking (*r* = 0.457, *p* < 0.01), human–AI collaboration quality (*r* = 0.620, *p* < 0.01), and research creativity (*r* = 0.456, *p* < 0.01). Existential dependence was also significantly positively correlated with critical thinking (*r* = 0.094, *p* < 0.01), human–AI collaboration quality (*r* = 0.258, *p* < 0.01), and research creativity (*r* = 0.148, *p* < 0.01). Overall, the correlation patterns were generally consistent with the proposed theoretical framework and provided a basis for the subsequent multivariate analyses.

**TABLE 2:** Descriptive statistics and correlation analysis.

Variable	M	SD	1	2	4	5	6
1. Functional dependence	3.591	0.725	1	1	1	1	1
2. Existential dependence	3.113	0.953	0.339**				
3. Critical thinking	3.765	0.772	0.457**	0.094**			
4. Human-AI collaboration quality	3.702	0.746	0.620**	0.258**	0.593**		
5. Research creativity	3.635	0.748	0.456**	0.148**	0.825**	0.573**	

N = 1157. **Indicates *p* < 0.01 (two-tailed); M, mean; SD, standard deviation.

### 4.3 Structural equation modeling results

To further validate the robustness of the proposed theoretical framework, structural equation modeling (SEM) was conducted using AMOS 24. A second-order structural model was specified in which GenAI dependence was modeled as a higher-order construct composed of functional dependence and existential dependence. Critical thinking and human-AI collaboration quality were specified as serial mediators linking GenAI dependence to research creativity.

The model demonstrated a satisfactory fit to the data: χ^2^/df = 7.541, CFI = 0.988, GFI = 0.981, and SRMR = 0.017, the model showed an overall acceptable fit to the data. Although the χ^2^/df ratio was relatively high, other fit indices indicated satisfactory model fit. These findings indicate that the proposed theoretical model adequately fits the observed data and supports the hypothesized structural relationships.

The standardized path coefficients are presented in Figure 1. GenAI dependence was positively associated with critical thinking (β = 0.492, *p* < 0.001), human–AI collaboration quality (β = 0.538, *p* < 0.001), and research creativity (β = 0.064, *p* < 0.05). Critical thinking was positively associated with human–AI collaboration quality (β = 0.348, *p* < 0.001) and research creativity (β = 0.731, *p* < 0.001). Human–AI collaboration quality was also positively associated with research creativity (β = 0.101, *p* < 0.001). These results were consistent with the proposed pattern of structural associations and provided statistical support for the subsequent examination of indirect pathways.

**FIGURE 1:**
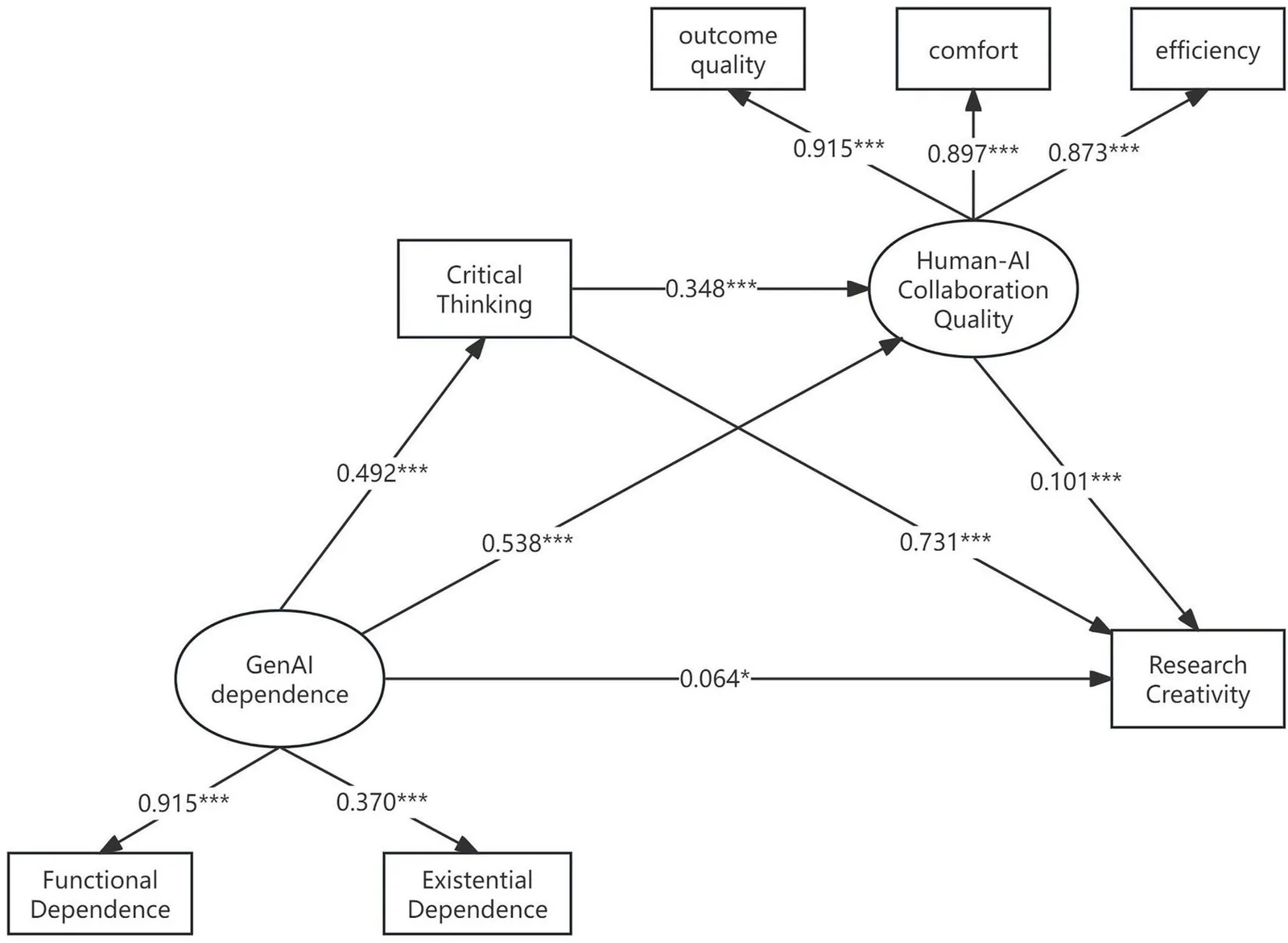
Structural equation model of the proposed framework.

Overall, the SEM results were consistent with the proposed serial mediation framework and supported further examination of the specific indirect associations using bootstrap analyses.

### 4.4 Serial mediation analysis

To further examine the differential associations of functional dependence and existential dependence with research creativity, the SPSS PROCESS macro (Model 6) was employed. Gender, degree type, grade level, and academic discipline were included as control variables. Bias-corrected bootstrap confidence intervals based on 5,000 resamples were used to estimate the indirect effects. Three specific indirect pathways were examined: Ind1, Dependence→ Critical Thinking→ Research Creativity; Ind2, Dependence→ Human–AI Collaboration Quality→ Research Creativity; and Ind3, Dependence→ Critical Thinking→ Human–AI Collaboration Quality→ Research Creativity. The results are reported in Tables 3, 4.

**TABLE 3:** Standardized path coefficients of the serial mediation models.

Path	Functional dependence	Existential dependence
Dependence→ critical thinking	0.464***	0.097**
Dependence→ human-AI collaboration quality	0.443***	0.205***
Critical thinking→ human-AI collaboration quality	0.388***	0.571***
Critical thinking→ research creativity	0.744***	0.755***
Human-AI collaboration quality→ research creativity	0.097***	0.111***
Direct effect	0.052*	0.045*
Total effect	0.457***	0.147***

Standardized coefficients are reported.

*p < 0.05,

**p < 0.01,

***p < 0.001.

**TABLE 4:** Bootstrap results for mediation effects.

Path/effect	Functional dependence	Existential dependence
Total indirect effect	0.4055 [0.3470, 0.4608]	0.1025 [0.0395, 0.1645]
Ind1: dependence→ critical thinking→ research creativity	0.3449 [0.2925, 0.3967]	0.0734 [0.0163, 0.1301]
Ind2: dependence→ human-AI collaboration quality→ research creativity	0.0431 [0.0182, 0.0703]	0.0229 [0.0117, 0.0364]
Ind3: dependence→ critical thinking→ human-AI collaboration quality→ research creativity	0.0175 [0.0072, 0.0301]	0.0062 [0.0012, 0.0128]

Bootstrap resampling = 5,000; 95% confidence intervals are reported; an effect is significant if the confidence interval does not contain zero.

#### 4.4.1 Functional dependence and research creativity

With functional dependence as the independent variable, the total effect on research creativity was significant (β = 0.457, *p* < 0.001), and the direct effect remained significant after including the mediators (β = 0.052, *p* < 0.05), indicating partial mediation. Functional dependence significantly and positively predicted critical thinking (β = 0.464, *p* < 0.001) and human-AI collaboration quality (β = 0.443, *p* < 0.001). Critical thinking significantly predicted both human-AI collaboration quality (β = 0.388, *p* < 0.001) and research creativity (β = 0.744, *p* < 0.001), while human-AI collaboration quality also positively predicted research creativity (β = 0.097, *p* < 0.001).

The bootstrap analysis showed that the total indirect effect was significant (effect = 0.4055, 95% CI [0.3470, 0.4608]). All three specific indirect pathways were significant: Ind1, through critical thinking alone (effect = 0.3449, 95% CI [0.2925, 0.3967]); Ind2, through human–AI collaboration quality alone (effect = 0.0431, 95% CI [0.0182, 0.0703]); and Ind3, sequentially through critical thinking and human–AI collaboration quality (effect = 0.0175, 95% CI [0.0072, 0.0301]). These results indicate that functional dependence was positively associated with research creativity both directly and through significant indirect pathways involving critical thinking and human–AI collaboration quality.

#### 4.4.2 Existential dependence and research creativity

With existential dependence as the independent variable, the total effect on research creativity was also significant (β = 0.147, *p* < 0.001), and the direct effect remained significant after controlling for the mediators (β = 0.045, *p* < 0.05). Existential dependence significantly predicted critical thinking (β = 0.097, *p* < 0.01) and human-AI collaboration quality (β = 0.205, *p* < 0.001). Critical thinking significantly predicted human-AI collaboration quality (β = 0.571, *p* < 0.001) and research creativity (β = 0.755, *p* < 0.001), while human-AI collaboration quality significantly predicted research creativity (β = 0.111, *p* < 0.001).

The total indirect effect was significant (effect = 0.1025, 95% CI [0.0395, 0.1645]). All three specific indirect pathways were also significant: Ind1, through critical thinking alone (effect = 0.0734, 95% CI [0.0163, 0.1301]); Ind2, through human–AI collaboration quality alone (effect = 0.0229, 95% CI [0.0117, 0.0364]); and Ind3, sequentially through critical thinking and human–AI collaboration quality (effect = 0.0062, 95% CI [0.0012, 0.0128]). These results indicate that existential dependence was also positively associated with research creativity through significant direct and indirect pathways involving critical thinking and human–AI collaboration quality, although the corresponding effect sizes were substantially smaller than those observed for functional dependence.

### 4.5 Differential effects analysis

To further compare the differential patterns associated with functional dependence and existential dependence, this study examined their total effects and specific indirect pathways. First, the total association between functional dependence and research creativity (β = 0.457) was substantially larger than that observed for existential dependence (β = 0.147), suggesting that functional dependence exhibited a stronger positive association with research creativity in the present sample. Because these estimates were obtained from separate mediation models, the difference is interpreted descriptively rather than as a formal statistical test of the difference between coefficients.

Second, the indirect association involving functional dependence was primarily concentrated in the pathway through critical thinking (Ind1 = 0.3449), accounting for approximately 85.0% of its total indirect effect. For existential dependence, the indirect effect through critical thinking was also the largest of the three specific indirect pathways (Ind1 = 0.0734), although its magnitude was considerably smaller than that observed for functional dependence. These results suggest that critical thinking represents an important statistical pathway linking both forms of GenAI dependence with research creativity, particularly in the functional dependence model.

Third, the serial indirect effect through critical thinking and human–AI collaboration quality was larger in the functional dependence model (Ind3 = 0.0175) than in the existential dependence model (Ind3 = 0.0062). Taken together, these patterns indicate that functional dependence showed stronger positive direct and indirect associations with research creativity than existential dependence, while both forms of dependence were associated with research creativity through critical thinking and human–AI collaboration quality.

## 5 Conclusion and discussion

### 5.1 Research conclusion

Based on survey data from 1,157 graduate students, this study examined the associations among GenAI dependence, critical thinking, human–AI collaboration quality, and self-reported research creativity, while further distinguishing functional dependence from existential dependence. The main findings are summarized as follows. First, the SEM results indicated that overall GenAI dependence was positively associated with critical thinking, human–AI collaboration quality, and research creativity. Critical thinking and human–AI collaboration quality were also positively associated with research creativity, providing support for the proposed pattern of structural associations. Second, the separate mediation analyses showed that both functional dependence and existential dependence were positively associated with research creativity. However, the magnitude of the total association was substantially larger for functional dependence than for existential dependence, indicating differentiated association patterns across the two forms of GenAI dependence. Third, critical thinking and human–AI collaboration quality each constituted significant indirect pathways linking both forms of GenAI dependence with research creativity. The indirect pathway through critical thinking was particularly prominent in the functional dependence model, accounting for approximately 85% of its total indirect effect. This finding highlights the close association between instrumental GenAI reliance, critical engagement, and research creativity. Fourth, critical thinking and human–AI collaboration quality jointly constituted a significant serial indirect pathway. The magnitude of this serial indirect association was larger in the functional dependence model than in the existential dependence model, suggesting that functional dependence was more strongly associated with a pattern involving critical evaluation, higher perceived human–AI collaboration quality, and research creativity. Overall, the findings demonstrate that GenAI dependence is not a homogeneous phenomenon. Functional and existential dependence show different association patterns with graduate students’ research creativity, while critical thinking and human–AI collaboration quality represent important cognitive and collaborative correlates within these relationships. These findings should nevertheless be interpreted as statistical associations rather than causal effects because all focal variables were assessed cross-sectionally.

### 5.2 Discussion

#### 5.2.1 Differential associations of functional and existential dependence with research creativity

This study found that both functional dependence and existential dependence positively predicted graduate students’ research creativity, although the positive effect of existential dependence was significantly weaker than that of functional dependence. The finding that existential dependence exerts a positive effect is somewhat inconsistent with previous studies suggesting that excessive AI dependence may lead to cognitive degradation and reduced independent thinking (Klingbeil et al., 2024; Dergaa et al., 2024; Gerlich, 2025). A possible explanation is that the cognitive stimulation generated through intensive AI interaction may partially offset the potential negative consequences associated with psychological attachment.

First, graduate students with higher levels of existential dependence tend to interact with GenAI more frequently and for longer periods of time. Such intensive engagement exposes them to a larger amount of information, more diverse perspectives, and richer interdisciplinary associations, thereby continuously providing novel cognitive input and creative stimulation. Even when some degree of uncritical acceptance exists, AI-generated content may still broaden graduate students’ cognitive horizons and facilitate the generation of creative ideas Second, the cognitive maturity of graduate students may buffers the negative effects of AI dependence. Compared with primary and secondary school students, graduate students generally possess stronger disciplinary foundations, metacognitive awareness, and academic judgment. Therefore, even when a certain degree of psychological dependence on AI develops, they are still capable of evaluating and filtering AI-generated outputs to some extent, selectively retaining valuable information while discarding low-quality content (Ringo, 2025). Third, the positive effect of existential dependence may also operate through an immersive cognitive coupling mechanism. Graduate students with existential dependence tend to regard GenAI as an external cognitive aid and become accustomed to engaging in continuous reasoning, hypothesis testing, and multi-turn dialogue. Although this interaction pattern is accompanied by a certain degree of psychological attachment, it may simultaneously create more opportunities for cognitive perturbation and intellectual collision, thereby stimulating novel associations and creative insights (Wu et al., 2026). The present study further found that the positive effect of existential dependence was substantially weaker than that of functional dependence. This finding suggests that psychologically attached dependence is not the optimal pathway for AI empowerment. Compared with existential dependence, functional dependence emphasizes active control, instrumental use, and cognitive agency during human–AI interaction. Therefore, the empowering effect of GenAI appears to depend not merely on the frequency of AI use, but more importantly on whether users maintain an active and reflective role in the interaction process. In other words, positioning GenAI as a tool-based cognitive assistant, rather than developing psychological attachment to it, represents a more effective approach to enhancing graduate students’ research creativity.

#### 5.2.2 The mediating role of critical thinking

This study found that the effect of functional dependence on critical thinking was substantially stronger than that of existential dependence. Moreover, the indirect effect of critical thinking in the relationship between functional dependence and research creativity accounted for approximately 85% of the total indirect effect. These findings provide strong support for the proposed research hypotheses and highlight the central role of critical thinking in the cognitive empowerment process of GenAI use. Graduate students with higher levels of functional dependence tend to maintain an active and reflective role during human–AI interaction. In the process of using AI for research assistance, they are required to continuously clarify task objectives, design effective prompts, evaluate the reliability of AI-generated outputs, identify potential errors and biases, and selectively revise or integrate AI-generated suggestions into their own reasoning processes. Such active monitoring and evaluative engagement essentially constitute systematic training in critical thinking, including analysis, reasoning, evaluation, and self-calibration (Zimmerman, 2000). Each effective interaction with AI may function as a micro-level exercise in critical thinking. Over time, these cognitively demanding interactions gradually strengthen graduate students’ critical thinking abilities, which can subsequently transfer to multiple dimensions of research creativity, such as identifying research gaps, evaluating evidence quality, and constructing innovative academic arguments (Spector and Ma, 2019; Liu and Wang, 2024). In contrast, although graduate students with existential dependence also interact with AI frequently, their interactions tend to be characterized by relatively passive acceptance and habitual reliance. Compared with functional dependence, existential dependence involves less proactive evaluation, critical revision, and independent judgment during the interaction process. Consequently, its capacity to strengthen critical thinking is relatively limited. This may explain why the mediating effect of critical thinking in the relationship between existential dependence and research creativity was substantially weaker than that observed in the functional dependence model. Overall, these findings suggest that the empowering effect of GenAI on research creativity depends not only on the frequency of AI use, but more importantly on whether users engage with AI in an active, reflective, and cognitively evaluative manner.

#### 5.2.3 The mediating role of human-AI collaboration quality

This study found that human-AI collaboration quality plays a significant mediating role in the relationship between GenAI dependence and research creativity. Regardless of whether the dependence takes a functional or existential form, higher levels of GenAI dependence were associated with better human–AI collaboration quality, which in turn positively promoted graduate students’ research creativity. Consistent with previous research, this finding reveals an important pathway through which GenAI facilitates creativity namely, by improving the interaction quality, coordination efficiency, and integration effectiveness between humans and AI systems, thereby enhancing research creativity (Vinchon et al., 2023; Huang, 2025).

The association between functional dependence and human–AI collaboration quality was stronger than that observed for existential dependence. One possible interpretation is that instrumental reliance is more compatible with active user control, iterative prompting, output evaluation, and strategic adjustment during human–AI interaction. Existential dependence may also be associated with perceived collaboration quality, potentially because greater reliance is accompanied by greater familiarity with AI-assisted work; however, the present study did not directly measure interaction frequency, accumulated experience, or familiarity, and this explanation therefore remains tentative.

More broadly, the findings suggest that productive human–AI collaboration may depend less on reliance itself than on whether users remain active participants in evaluating and integrating AI-generated outputs. From this perspective, maintaining human cognitive agency and reflective oversight may be particularly important when GenAI becomes deeply embedded in graduate research activities.

#### 5.2.4 The serial mediating role of critical thinking and human-AI collaboration quality

This study found that critical thinking and human-AI collaboration quality jointly form a significant serial mediation pathway linking GenAI dependence to graduate students’ research creativity, and this pathway was particularly pronounced in the functional dependence model. The theoretical significance of this finding lies in its clarification of the complete psychological mechanism through which GenAI affects creativity. Specifically, the use of GenAI first reshapes individuals’ cognitive patterns; these enhanced cognitive patterns subsequently improve the quality of human-AI interaction; and ultimately, high-quality collaboration between humans and AI promotes creative research outcomes. The positive influence of critical thinking on human-AI collaboration quality verifies the logic that thinking quality is a prerequisite for high-quality collaboration. Only graduate students with critical thinking can accurately assess the value of AI outputs, flexibly adjust interaction strategies, and develop calibrated trust, thereby establishing a truly complementary collaborative relationship with AI rather than a one-sided dependent relationship (Raitskaya and Tikhonova, 2025; Hou et al., 2026). In addition, this deep collaboration relationship further stimulates graduate students’ innovative potential through the mechanisms of cognitive extension and creative perturbation (Clark and Chalmers, 1998). AI not only expands individuals’ access to information and perspectives but also continuously introduces novel ideas and unexpected associations during interaction, thereby encouraging researchers to break through conventional thinking patterns and generate more original research insights. Compared with existential dependence, functional dependence is more likely to activate this complete transmission chain from cognitive enhancement to collaborative optimization, ultimately producing a stronger positive effect on research creativity.

### 5.3 Practical implications

The findings have several implications for graduate education and institutional AI literacy initiatives. First, universities should avoid treating GenAI dependence as a uniformly desirable or undesirable phenomenon. The distinction between functional and existential dependence suggests that AI education should focus not simply on reducing or increasing GenAI use, but on promoting purposeful, task-oriented, and critically supervised use. Graduate students should be encouraged to position GenAI as a cognitive support tool while retaining responsibility for problem formulation, evidence evaluation, methodological decisions, and final scholarly judgments.

Second, critical thinking should be incorporated more explicitly into graduate AI literacy training. Rather than focusing primarily on prompt engineering or technical proficiency, training programs could include verification of AI-generated claims, identification of hallucinations and bias, comparison of alternative outputs, source checking, and justification of decisions to accept, revise, or reject AI-generated suggestions. Such practices may help students maintain cognitive agency as GenAI becomes increasingly embedded in research workflows.

Third, institutions and supervisors should promote reflective human–AI collaboration while remaining attentive to signs of deeper psychological reliance. Practical guidance may encourage students to document how AI-generated content is evaluated and modified, periodically conduct important research tasks independently of GenAI, and use human supervision or peer discussion for high-stakes academic decisions. The objective should therefore be neither unconditional adoption nor categorical avoidance of GenAI, but the development of calibrated, transparent, and human-directed patterns of AI-assisted research.

## 6 Research limitations and future prospects

First, this study relied on cross-sectional survey data, which prevents strong causal inference and does not establish the temporal ordering among GenAI dependence, critical thinking, human–AI collaboration quality, and research creativity. Although SEM and serial mediation analyses were used to examine theoretically specified associations, the estimated indirect effects should be interpreted as statistical indirect associations rather than evidence of causal mediation. Reverse causality remains possible; for example, graduate students with stronger critical thinking or higher research creativity may be more likely to use GenAI strategically. In addition, unmeasured variables such as prior AI experience, research self-efficacy, academic ability, supervisory support, or previous research productivity may contribute to the observed associations. Future research should employ longitudinal, cross-lagged, experimental, or intensive longitudinal designs to examine temporal ordering and causal processes more rigorously. Second, participants were recruited through online convenience sampling using university-based WeChat groups and social media platforms. This recruitment strategy may introduce self-selection bias and limits the representativeness of the sample. Graduate students from different institutional, disciplinary, regional, and cultural contexts may display different patterns of GenAI use and dependence. Future studies should employ probability-based or stratified sampling where feasible and test the generalizability of the findings across institutions, disciplines, regions, and countries. Third, all focal constructs were assessed using self-report measures, which may increase shared-method variance and may not fully capture actual research behavior or creative performance. Research creativity, in particular, reflects participants’ perceptions of their own creativity rather than independently evaluated research output. Future studies could combine self-report measures with objective or multi-source indicators, such as blind expert ratings of research proposals or manuscripts, originality ratings of research ideas, publication or patent outputs, supervisor assessments, behavioral traces of GenAI use, prompt-interaction logs, and longitudinal indicators of research productivity and creativity. Fourth, although the present study identified positive associations between both forms of GenAI dependence and self-reported research creativity, these findings should not be interpreted as implying that greater dependence is uniformly beneficial. Existential or excessive dependence may carry important risks, including cognitive offloading, reduced originality, algorithmic conformity, weakened independent reasoning, and diminished confidence in completing research tasks without AI assistance. Future research should examine potential nonlinear or threshold effects and identify conditions under which GenAI reliance shifts from productive support to maladaptive dependence. Finally, this study conceptualized GenAI dependence as consisting of functional dependence and existential dependence; however, the boundaries and developmental trajectories of these two forms of dependence may continue to change alongside technological advancement and increasing human-AI integration. Future research may further explore the dynamic evolution of human-AI relationships and investigate how different patterns of AI dependence interact with individual traits, educational environments, and disciplinary contexts to shape graduate students’ long-term cognitive development and research innovation. Overall, future research should continue to investigate the complex and evolving mechanisms through which generative AI reshapes graduate students’ cognition, collaboration, and creativity in academic contexts, thereby providing a stronger theoretical and empirical foundation for the responsible integration of AI into higher education and research practices.

## Data Availability

The raw data supporting the conclusions of this article will be made available by the authors, without undue reservation. Further inquiries can be directed to the corresponding author/s.
